# Enhancing Soybean (*Glycine max* L. Merr) Heat Stress Tolerance: Effects of Sowing Date on Seed Yield, Oil Content, and Fatty Acid Composition in Hot Climate Conditions

**DOI:** 10.1002/fsn3.4690

**Published:** 2024-12-19

**Authors:** Seyed Ahmad Kalantar Ahmadi, Jahanfar Daneshian

**Affiliations:** ^1^ Department of Agronomy and Horticultural Science, Safiabad Agricultural and Natural Resources Research and Education Center Agricultural Research, Education and Extension Organization (AREEO) Dezful Iran; ^2^ Seed and Plant Improvement Institute Agricultural Research, Education and Extension Organization (AREEO) Dezful Iran

**Keywords:** genotype, protein, seed‐filling, soybean, temperature

## Abstract

High temperatures can impede the growth and development of soybean plants, resulting in decreased yield and seed quality. Heat‐induced damage can be mitigated by adjusting sowing date and selecting genotypes that are suitable for cultivation in hot climates. A 2‐year (2017–2018) field experiment was conducted at Safiabad Agricultural and Natural Resources Research and Education Center, employing a split‐plot design with three replications. The main plots were assigned three different sowing dates (June 22, July 6, and July 21), while the subplots featured eight soybean genotypes (SF1, SF2, SF3, SK93, M13, SG4, SG5, and Salend) belonged to IV to VI maturity groups. Temperature affected the fatty acid composition across all genotypes. Planting soybeans on June 22 and July 6 resulted in a 16% and 8% decrease in seed yield, respectively, compared to planting on July 21 over 2 years of experiments. SK93 exhibited the highest oil content (25.59%) when sown on the third date (July 21), whereas the SF3 genotype planted on June 22 displayed the lowest oil content (18.68%). Based on our findings, a decrease of approximately 0.33% in oil content and a 0.7% increase in protein content were observed with a one‐degree temperature rise from 33°C during the seed‐filling period. When the temperature ranged between 36°C and 38°C, the highest seed yield (2665–3008 kg.ha^−1^) was obtained, whereas the lowest seed yield (1940 kg.ha^−1^) occurred at 41.60°C. Delaying planting led to a higher seed yield (19.72%) and enhanced seed oil content (11.54%). The indeterminate growth genotype SK93 consistently showed the highest average seed yield (3231 kg.ha^−1^) over the 2‐year experiment, exceeding other genotypes.

## Graphical abstract

Seed yield, oil content, and protein content of soybean genotypes significantly changed under different temperature regimes. Temperature during the seed‐filling period affects the composition of fatty acids of soybean genotypes. High temperature during the seed‐filling period altered unsaturated fatty acid composition by increasing oleic acid and decreasing linoleic and linolenic acids. Soybean genotypes had various reaction to high temperature due to changing of sowing date.

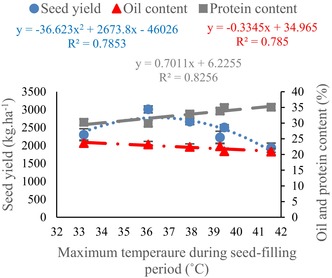

## 1 Introduction

Climate change is characterized by significant shifts in weather patterns, particularly in rainfall and temperature (Malhi, Kaur, and Kaushik 2021). The global average temperature is on an upward trajectory, anticipated to increase by 2°C by 2100 (Malhi, Kaur, and Kaushik 2021). Crops are experiencing increased abiotic stresses due to the current warming trend as well as altered climatic patterns (Shahzad, Ullah, and Dar 2021). Furthermore, the frequency and intensity of prolonged summer heat waves are expected to rise (Balfagón et al. 2022; Ullah et al. 2022; Sun et al. 2022).

Elevated temperatures disrupt plant growth and development, leading to reduced yields across various crops (Malhi, Kaur, and Kaushik 2021; Kalantarahmadi and Daneshian 2023; Jianing et al. 2022). Soybean is particularly vulnerable to these effects (Carrera and Dardanelli 2017; Allen et al. 2018). Soybean, a cornerstone of global food production, ranks as the second most important oilseed crop (Carrera and Dardanelli 2017), which is a primary source of protein worldwide (Bihter et al. 2017). Soybean seeds are rich in nutrients, boasting approximately 40% protein, 20% oil, and 33% carbohydrates (Akond et al. 2018; Alsajri et al. 2020).

For crops to be produced optimally in any region, there needs to be enough growth and development opportunities and adaptations to the local climate (Kalantar Ahmadi 2022; Kalantar Ahmadi and Eyni‐Nargeseh 2023). Soybean is under threat from climate change. A global temperature increase of 1°C over the past century (Zhao et al. 2017) has already resulted in a 17% reduction in soybean seed yield (SY; Lobell and Asner 2003). Rising temperatures are known to negatively affect the quantity and quality properties of soybean (Alsajri et al. 2020) such as yield, oil content (Cohen et al. 2021; Jianing et al. 2022), fatty acid composition, and protein content (Alsajri et al. 2020; Bihter et al. 2017; Mishra and Cherkauer 2010). Heat stress during the vegetative stage can hinder photosynthesis, reduce leaf area index, and decrease plant growth. However, the most damaging effect occurs during the reproductive phase, leading to flower abortion, reduced pollen viability, and ultimately a decline in SY, seed size, and oil content (Jumrani and Bhatia 2018; Jumrani, Bhatia, and Pandey 2017; Iovane and Aronne 2022). Exposure to high temperatures (39°C day and 29°C night) was shown to significantly reduce pollen germination and viability (Djanaguiraman, Prasad, and Schapaugh 2013). Furthermore, heat stress during the grain‐filling stage affects the composition of seeds (Song et al. 2016; Nakagawa et al. 2020).

Soybean plants are highly dependent on daylight for photosynthesis, accumulating energy reserves during the day which are then utilized for growth and development throughout the night (Xu et al. 2021). While average daily temperatures are important, research showed that temperature fluctuations, particularly those occurring at night, have a significant impact on soybean seed composition, specifically oil and protein content (Song et al. 2016).

Soybean oil contains three unsaturated fatty acids (oleic, linoleic, and linolenic) and two saturated fatty acids (palmitic and stearic). Research has shown that high temperatures can alter the proportions of these fatty acids. Studies by Oliva, Shannon, and Sleper (2006), Bellaloui, Reddy, and Mengistu (2015), and Matías et al. (2021) indicated that elevated temperatures lead to a decrease in linoleic and linolenic acid levels, while oleic acid concentration increases. This effect is dependent on the intensity and duration of the high temperature exposure, as outlined by Kalantarahmadi and Daneshian (2023). A soybean genotype with stable oleic acid and linolenic acid under different environmental conditions may also be considered a favorable source with suitable fatty acid composition because its genetic characteristics affect unsaturated fatty acids differently (Oliva, Shannon, and Sleper 2006; Bellaloui, Reddy, and Mengistu 2015). Alsajri et al. (2020) showed that heat stress during the grain‐filling period can increase the levels of saturated fatty acids in soybeans. Furthermore, the amount of changes in saturated fatty acids depends on the genotype, environmental conditions, and their interaction (Hou et al. 2006). This increase is likely due to the impact of high temperatures on the activity of enzymes responsible for fatty acid accumulation and conversion, as described by Alsajri et al. (2020). High temperatures can have broader effects on soybean plants, leading to changes in protein content, as reported by Nakagawa et al. (2020) and Yang et al. (2023), as well as damage to the vacuole structure and cellular membrane, which serve as storage sites for proteins, as highlighted by Krishnan et al. (2020).

Soybean plants employ different mechanisms such as gas exchange regulation, reduced leaf surface area to maintain photosynthetic activity for increased seed weight, preservation of pollen germination potential (Poudel et al. 2023), shortened seed‐filling period, and increased filling rate to enhance heat stress tolerance (Pradhan et al. 2012). Heat stress during flowering and podding stages causes irreversible damage to soybeans (Buezo et al. 2019). Selecting heat‐tolerant genotypes and genetic diversity (Ortiz et al. 2022) can improve SY (Bheemanahalli et al. 2022; Poudel et al. 2023).

Proper agronomic management, hormone application, growth regulators, and signaling molecules can improve heat tolerance mechanisms (Nahar, Hasanuzzaman, and Fujita 2016). Abiotic stresses such as drought and heat have an impact on gene expression patterns and protein synthesis (Manan and Zhao 2020). Osmotic stress induced by drought or heat (Kim et al. 2010) increases ABA synthesis, leading to changes in stomatal closure, gene expression, and physiological adaptation responses. Heat‐tolerant genes influence stress tolerance via gene expression and hormone regulation (Manan and Zhao 2020).

Soybean stands as a significant source of both oil and protein, making it a highly valuable commodity worldwide. Its cultivation also plays a vital role in improving soil fertility. Anticipating a surge in demand for oil and protein, soybean cultivation is projected to expand. In order to maximize yield, protein and oil content, and fatty acid composition, effective crop management practices are crucial, including selection of the appropriate sowing date and genotype. The agricultural sector in Iran, particularly in the hot and arid Khuzestan province, was significantly affected by increasing temperatures in recent years. There is a constant challenge in this region with finding appropriate soybean genotypes and the optimal sowing dates for mitigating heat stress during the reproductive stages. This research aims to examine the effects of high temperatures, as affected by sowing date, on the yield and seed composition of various soybean genotypes, offering valuable insights for improving soybean cultivation in challenging environments.

## 2 Material and Methods

### 2.1 Experimental Site

**FIGURE 1:**
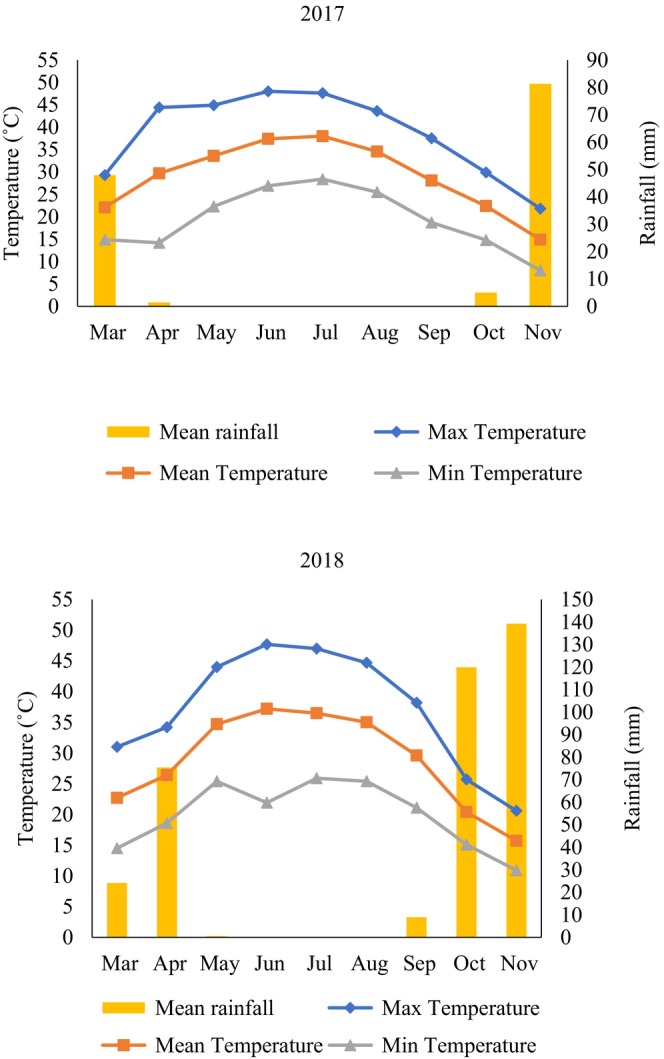
Climatic parameters (air maximum, minimum, and mean of temperature and rainfall) in the experiment site (2017 and 2018).

This field study was carried out at the Safiabad Agricultural Research, Education, and Natural Resources Center in Khuzestan province, Iran (32°16′ N, 48°26′ E, 82.9 m above sea level). The experiment spanned two growing seasons, 2017 and 2018. Table 1 provides detailed information about the soil characteristics at the experimental site. Figure 1 presents the meteorological data collected during the two cultivation years, including average, minimum, and maximum temperatures, as well as rainfall. Tables 2 and 3 detail the temperature fluctuations during the reproductive and seed‐filling stages, respectively. The number of days between R5–R7 was considered as the length of seed‐filling period. Days from R1 to R8 were recorded as the length of the reproductive stage (Fehr and Caviness 1977).

**TABLE 1:** Physical and chemical properties of soil used in the experiment.

Year	Soil texture	OC (%)	N (%)	P (ppm)	K (ppm)	pH	EC (ds/m)
2017	Clay‐Loam	0.62	0.082	8.5	180	7.66	0.54
2018	Clay‐Loam	0.64	0.067	9.1	184	7.61	0.56

Abbreviations: EC, electrical conductivity; K, potassium; N, nitrogen; OC, organic carbon; P, phosphorus; pH, potential of hydrogen.

**TABLE 2:** Temperature during the reproductive period of soybean genotypes in different sowing dates (2017–2018).

Sowing date	Genotypes	2017	Mean temperature (°C)	Max temperature (°C)	2018	Mean temperature (°C)	Max temperature (°C)
June 22	SF1	Aug. 12–Oct. 22	32.38	41.56	Aug. 26–Oct. 30	31.02	39.7
	SF2	Aug. 10–Oct. 22	32.32	41.5	Aug. 29–Oct. 30	30.74	39.38
	SF3	Aug. 9–Oct. 12	33.33	42.47	Aug. 18–Oct. 30	32.38	41.58
	SK93	Aug. 18–Oct. 21	32.02	41.23	Sep. 1–Oct. 29	30.7	39.29
	M13	Aug. 12–Oct. 22	32.38	41.56	Sep. 1–Oct. 30	31.51	39.08
	SG4	Aug. 13–Oct. 22	32.32	41.5	Aug. 31–Oct. 30	30.42	38.97
	SG5	Aug. 14–Oct. 22	32.23	41.42	Aug. 31–Oct. 30	30.42	38.97
	Salend	Aug. 13–Oct. 24	32.39	41.27	Sep. 2–Oct. 30	30.26	38.8
July 6	SF1	Aug. 24–Oct. 12	32.34	41.56	Sep. 6–Nov. 4	29.43	37.47
	SF2	Aug. 24–Oct. 12	32.34	41.56	Sep. 7–Nov. 4	29.35	37.75
	SF3	Aug. 22–Oct. 21	31.46	40.69	Sep. 1–Oct. 28	30.92	39.55
	SK93	Aug. 28–Nov. 3	29.78	38.83	Sep. 8–Nov. 1	29.68	38.2
	M13	Aug. 25–Nov. 9	29.05	37.94	Sep. 8–Oct. 31	29.8	38.31
	SG4	Aug. 25–Nov. 9	29.05	37.94	Sep. 8–Nov. 6	29.08	37.29
	SG5	Aug. 24–Nov. 7	29.29	38.23	Sep. 9–Nov. 7	28.87	37
	Salend	Aug. 26–Nov. 10	28.74	37.54	Sep. 8–Nov. 5	29.96	37.55
July 21	SF1	Sep. 21–Nov. 21	25.36	33.84	Sep. 15–Nov. 10	27.7	35.48
	SF2	Sep. 21–Nov. 21	25.61	33.84	Sep. 17–Nov. 10	24.43	35.12
	SF3	Sep. 20–Nov. 19	25.07	34.13	Sep. 13–Nov. 2	29.04	37.35
	SK93	Sep. 22–Nov. 23	23.86	33.38	Sep. 16–Oct. 31	28.9	37.14
	M13	Sep. 27–Dec. 2	24.09	32.26	Sep. 18–Nov. 1	28.16	36.28
	SG4	Sep. 26–Dec. 2	24.05	32.55	Sep. 16–Nov. 4	28.07	35.99
	SG5	Sep. 26–Dec. 1	24.94	32.47	Sep. 17–Nov. 4	27.92	35.8
	Salend	Sep. 23–Nov. 23	26.14	33.24	Sep. 17–Nov. 6	27.68	35.48

**TABLE 3:** Temperature during the grain‐filling period of soybean genotypes in different sowing dates (2017–2018).

Sowing date	Genotypes	2017	Mean temperature (°C)	Max temperature (°C)	2018	Mean temperature (°C)	Max temperature (°C)
June 22	SF1	Sep. 3–Oct. 3	32.9	42.17	Sep. 12–Oct. 9	32.54	41.84
	SF2	Sep. 8–Oct. 5	32.23	41.35	Sep. 13–Oct. 11	32.33	41.6
	SF3	Aug. 24–Sep. 25	34.19	43.63	Aug. 28–Sep. 25	34.58	44.16
	SK93	Sep. 7–Oct. 9	31.43	40.35	Sep. 15–Oct. 12	31.57	40.69
	M13	Sep. 8–Oct. 7	31.66	40.75	Sep. 16–Oct. 11	31.65	40.84
	SG4	Sep. 7–Oct. 10	31.66	40.77	Sep. 15–Oct. 11	31.76	40.98
	SG5	Sep. 9–Oct. 8	31.5	40.77	Sep. 17–Oct. 11	31.52	40.66
	Salend	Sep. 7–Oct. 13	30.92	40.28	Sep. 16–Oct. 14	31.42	40.22
July 6	SF1	Sep. 13–Oct. 9	30.76	39.99	Sep. 20–Oct. 11	31.01	40.5
	SF2	Sep. 17–Oct. 12	30.04	39.39	Sep. 24–Oct. 17	30.03	38.97
	SF3	Sep. 12–Oct. 5	31.84	41.11	Sep. 16–Oct. 8	32.09	41.36
	SK93	Sep. 20–Oct. 19	28.59	38.3	Sep. 26–Oc. 23	29.19	37.53
	M13	Sep. 19–Oct. 17	28.62	38.06	Sep. 26–Oct. 22	29.47	38.01
	SG4	Sep. 18–Oct. 18	29.27	38.7	Sep. 21–Oct. 18	29.87	38.5
	SG5	Sep. 23–Oct. 14	29.18	38.68	Sep. 26–Oct. 19	29.9	38.5
	Salend	Sep. 19–Oct. 15	29.5	39.03	Sep. 23–Oct. 21	29.8	38.43
July 21	SF1	Oct. 1–Oct. 20	21.68	28.99	Oct. 5–Oct. 24	28.29	36.06
	SF2	Oct. 1–Oct. 20	27.06	36.41	Oct. 6–Oct. 24	28.15	35.82
	SF3	Sep. 22–Oct. 16	28.67	38.54	Sep. 26–Oct. 18	29.96	38.69
	SK93	Sep. 28–Oct. 27	26.95	36.22	Oct. 2–Oct. 28	27.4	34.88
	M13	Oct. 7–Oct. 24	25.93	35.28	Oct. 12–Oct. 29	25.48	32.36
	SG4	Oct. 2–Oct. 24	26.22	35.57	Oct. 5–Oct. 27	27.63	35.07
	SG5	Oct. 2–Oct. 24	26.22	35.57	Oct. 28–Nov. 6	26.91	34.23
	Salend	Oct. 3–Oct. 22	26.45	35.72	Oct. 7–Oct. 26	27.55	35.13

### 2.2 Experimental Design

This study employed a split‐plot design within the framework of a randomized complete block design, incorporating three replications. The main plot treatments comprised three sowing dates (June 22, July 6, and July 21), and the subplot treatments involved eight soybean genotypes (SF1, SF2, SF3, SK93, M13, SG4, SG5, and Salend). Saland is one of the cultivars that has been introduced for cultivation in the hot areas of the south of Iran. It can provide a higher SY than the informer cultivars and help tolerate high temperature stress. The results of previous experiments have shown that planting soybeans until June causes the periods of flowering and seed‐filling to collide with high air temperatures. High temperature stress causes the flowers to drop and seeds to abort in the pod. All these events cause the grain yield to decrease significantly. With the delay of planting in July, the occurrence of moisture‐bearing stages collides with lower temperature and increases the yield. However, the tolerance of soybean genotypes is different (Kalantar Ahmadi and Daneshian 2014; Kalantar Ahmadi, Eslamizadeh, and Ghodrati 2018). Given that the treatment of sowing date requires larger plots, the split‐plot design was chosen and the sowing date treatment was applied to main plots. Additionally, ease of experiment execution and the possibility of providing a more suitable implementation are considered advantages of this design. Characteristics of studied genotypes are shown in Table 4. Salend cultivar was considered as control genotype. Phonologic stages of genotypes were recorded by the Fehr and Caviness method (Fehr and Caviness 1977).

**TABLE 4:** Origin, maturity group, and growth type of soybean genotypes.

Soybean genotypes	Maturity group	Growth type	Origin
SF1	5	Indeterminate	Iran
SF2	5	Indeterminate	Iran
SF3	4	Indeterminate	Iran
SK93	5	Indeterminate	Iran
M13	5	Indeterminate	Iran
SG4	6	Indeterminate	Iran
SG5	6	Indeterminate	Iran
Salend (control)	6	Determinate	Iran

### 2.3 Experiment Details

The experimental layout was designed with a ridge spacing of 75 cm, where each ridge accommodated two sown rows, maintaining a plant density of 40 plants per square meter. The recommended fertilizer application rates were 45 kg ha^−1^ of nitrogen (N), 32 kg ha^−1^ of phosphorus (P), and 83 kg ha^−1^ of potassium (K). Prior to planting, all of the potassium sulfate and triple superphosphate, along with one‐third of the urea, were applied. The remaining two‐thirds of the urea were split into two equal applications at the R1 (beginning of bloom) and R3 (beginning of pod) stages. To enhance nitrogen fixation, soybean seeds were inoculated with *Bradyrhizobium japonicum* , obtained from the Research Institute of Soil and Water in Karaj, Iran. A 2% sucrose solution was added as a bacterial protectant. However, due to the lack of symbiotic nitrogen fixation, nitrogen fertilizer was still applied. Weed management was executed by applying 2 L ha^−1^ of treflan (trifloralin) before planting and bentazon 2.5 L ha^−1^ at the V4 stage, supplemented by manual weeding throughout the growth period as necessary.

### 2.4 SY, Oil Content, and Fatty Acid Composition Assessment

At physiological maturity (BBCH scale: 89), SY was determined by harvesting four center rows from each plot, covering an area of 6 m^2^. To measure oil content, 30 g of seeds from each plot (three replications) was collected and analyzed using a German Broker Brand minispec mq20 model Nuclear Magnetic Resonance (NMR) instrument (International Standard ISO 5511 1992). For this purpose, after the daily calibration of the device with the daily check sample and also calibration with prepared standard soybean samples, a certain amount of sample was transferred to the cell of the NMR device. The cell containing the sample was placed in its special position, and the oil percent was read on the monitor in < 1 min.

For the measurement of saturated and unsaturated fatty acids, the gas chromatography method was utilized. Initially, the oil from the samples was extracted using the Soxhelt method (Azadmard‐Damirchi, Savage, and Dutta 2005). Subsequently, the fatty acids were converted to fatty acid methyl esters using sodium hydroxide and boron trifluoride (Savage, Mcneil, and Dutta 1997) and then injected into the gas chromatograph. The gas chromatograph used was a Varian brand, model CP‐3800, equipped with a flame ionization detector, a split injector, and a capillary column. The fatty acids were identified by comparing the retention times of the peaks obtained in the gas chromatogram of the sample with the Sigma‐Aldrich standards of fatty acid methyl esters and quantified as peak area percentage. Seed protein content was measured using the Kjeldahl method (Association of Official Analytical Chemists (AOAC) 1980).

### 2.5 Statistical Analysis

To ensure reliable data analysis, the homogeneity of variance across experimental groups was evaluated using Bartlett's test. Subsequently, a combined analysis of variance was conducted using SAS software (version 9.2). The GGE biplot software facilitated the creation of a visual representation of the average performance of soybean genotypes in terms of SY stability. Data analysis was performed using SAS software (version 9.2). Mean comparisons were conducted using the least significant difference test at a significance level of 0.05. The relationship between measured traits and maximum temperature during grain‐filling was examined using regression analysis based on the means of interactions between year and sowing date. Graphs were generated using Excel software.

## 3 Results

Analysis of variance showed significant year effects on SY, oleic acid, linoleic acid, linolenic acid, and stearic acid. Sowing date and genotype demonstrated statistically significant impacts on all studied traits. The interaction between year and sowing date significantly affected oleic acid, palmitic acid, and stearic acid levels. Similarly, the interaction of year and genotype significantly affected SY, oil content, and stearic acid. Based on the combined analysis, it was found that sowing date and genotype interaction was statistically significant with regard to SY, oil content, oleic acids, linoleic acids, and palmitic acids. Furthermore, SY, oleic acid, and palmitic acid were influenced by the combined effects of year, sowing date, and genotype (Table 5).

**TABLE 5:** Combined analysis of variance for studied traits as affected by sowing date (S) and genotype (G) during 2 years (2017 and 2018) in Dezful, Iran.

S.O.V	*df*	Seed yield	Protein content	Oil content	Oleic acid	Linoleic acid	Linolenic acid	Palmitic acid	Stearic acid
Y	1	**	ns	ns	**	*	**	ns	**
E	4	—	—	—	—	—	—	—	—
S	2	**	**	**	**	**	**	**	**
Y × S	2	ns	ns	ns	**	ns	ns	**	**
E	8	—	—	—	—	—	—	—	—
G	7	**	**	**	**	**	**	**	**
Y × G	7	**	ns	**	ns	ns	ns	ns	**
S × G	14	**	ns	**	**	*	ns	**	ns
Y × S × G	14	**	ns	ns	**	ns	ns	**	ns
E	84	—	—	—	—	—		—	—
CV%		10.48	8.16	3.63	4.29	3.38	17.07	4.94	6.8

*Note:* * and ** significant at 5% and 1% probability level, respectively.

Abbreviations: CV, coefficient of variation; *df*, degree of freedom; E, error; G, genotype; ns, not significant; S, sowing date; Y, year.

### 3.1 SY

**FIGURE 2:**
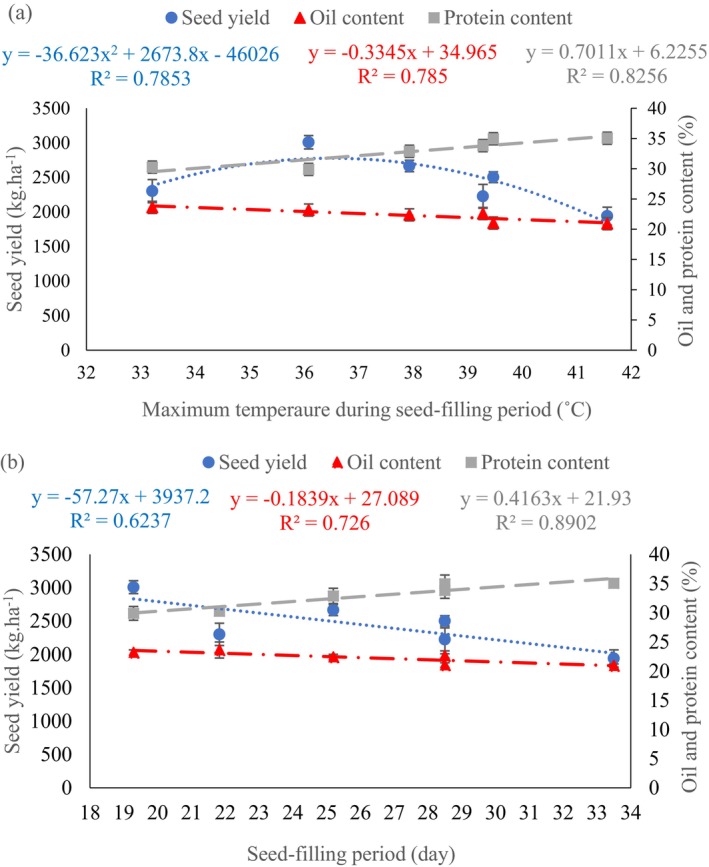
Regression relationship of seed yield, oil content, and protein content with maximum temperature during the seed‐filling period (a) and length of the seed‐filling period (b). Means of interaction year × sowing date were used (Each point shows mean three replications).

The mean comparison of two‐way interaction of sowing date × genotype showed that the highest SY (3473.9 kg.ha^−1^) appointed to SK93 genotype at the second sowing date (July 6) in the first year. The maximum SY (3559.7 kg.ha^−1^) belonged to SK93 genotype at the third sowing date (July 21) in the second year (Table 6). The lowest SY was detected to SF2 genotype at the first sowing date (June 22) in both years (968.9 kg.ha^−1^ and 2080 kg.ha^−1^ in the first and second years, respectively) (Table 6). A negative regression relationship was observed between SY and maximum temperature during the grain‐filling period (Figure 2a), indicating that higher temperatures negatively impacted SY. The highest SY (3008 kg.ha^−1^) was obtained at an approximate maximum temperature of 36°C. Moreover, a negative regression was found between SY and the length of the seed‐filling period (Figure 2b), demonstrating that longer seed‐filling periods resulted in decreased yields.

**TABLE 6:** Mean comparisons of the two‐way interaction of sowing date × genotype on seed yield, oleic acid, and palmitic acid in each year of experiment (2017 and 2018).

Sowing date	Genotype	Seed yield (kg.ha^−1^)		Oleic acid (%)		Palmitic acid (%)	
		2017	2018	2017	2018	2017	2018
June 22	SF1	1747.2hi	2416.7j–n	21.35h–k	20.21d–g	12.61a	13.34a
	SF2	968.9k	2080n	22.12g–j	20.92d–f	11.47c–f	13.17a
	SF3	1520ij	2304.7k–n	22.34f–i	23.96c	11.34d–g	10.86ef
	SK93	2660.9d–f	3022.3c–f	29.06a	31.31a	11.2e‐h	12.75a–c
	M13	2380.6fg	2941d–g	26.91b	26.83b	11.08e–h	12.86ab
	SG4	2655.6d–f	2100n	24.58cd	25.25bc	12.16bc	13.16a
	SG5	1705.4hi	2330.7k–n	23.64d–f	24.32c	12.12b–d	13.22a
	Salend	1888.9hi	2825e–i	22.66f–h	23.91c	13a	11.96b–e
July 6	SF1	1052.5jk	2792.6e–j	20.01k	19.18gh	11.79c–e	12.87a–d
	SF2	1452.8ij	2492.2i–m	20.96i–k	17.72h	10.9f–j	11.87b–e
	SF3	1505.6ij	2412.2k–n	21.37h–k	18.85gh	10.77f–k	11.79b–e
	SK93	3473.9a	3363.3a–c	27.17b	24.74c	10.43h–m	11.6c–e
	M13	2836.2c–f	2652f–k	25.94bc	24.49c	10.39i–m	11.07e
	SG4	3011.1a–d	2543.3h–l	23.44d–g	21.74d	10.77f–k	11.26de
	SG5	2027.8gh	2248.9mn	22.25g–i	20.23d–g	10.34i–m	11.29de
	Salend	2444.4fg	2817.3e–i	22.14g–j	21.61d	12.26a–c	11.68c–e
July 21	SF1	1483.3ij	2912.2e–h	18.53L	19.12gh	10.58g–l	9.16g–i
	SF2	1520ij	2594.4g–l	20.12k	17.88h	10.27j–m	9.77fg
	SF3	1561.1hi	2148.9mn	20.51k	17.85h	9.99k–m	9.08g–i
	SK93	3306.7a–c	3559.7a	24.05de	21.04de	9.91lm	8.13ij
	M13	2980.6b–e	3315.7a–d	22.97e–g	19.65e–g	9.73m	7.57j
	SG4	3355.6ab	3017.8c–f	21.24i–k	19.27f–h	10k–m	8.5h–j
	SG5	1763.5hi	3435.6ab	20.8jk	19.15gh	9.69m	8.37h–j
	Salend	2522.2ef	3084b–e	20.28k	17.91h	8.88n	9.48gh
LSD		477.11	379.02	1.37	1.71	0.79	1.14

*Note:* Means in each column, followed by similar letter(s) are not significantly different at the 5% probability level using the LSD test.

### 3.2 Oil Content

The interaction analysis between sowing date and genotype showed that the SK93 genotype consistently exhibited the highest oil content across all sowing dates, reaching a maximum of 25.59% on 21 July. The SF3 genotype, sown on 22 June, displayed the lowest oil content at 18.68% (Table 7). Regression analysis revealed a linear negative relationship between oil content and increasing temperature (Figure 2a). In addition, there was a negative correlation between seed‐filling duration and oil content (Figure 2b). The highest (24.59%) and the lowest (19.52%) oil content belonged to SK93 and SF3 genotypes, respectively (Figure 5a).

**TABLE 7:** Mean comparisons of the two‐way interaction of sowing date × genotype on seed yield, oleic acid, and palmitic acid during the 2 study years (2017 and 2018).

Sowing date	Genotype	Oil content (%)	Linoleic acid (%)
June 22	SF1	19.65jk	48.54jk
	SF2	19.83ij	48.42jk
	SF3	18.68k	48.24k
	SK93	23.37d	51.31g–i
	M13	20.69g–i	53.98a–e
	SG4	21.98ef	51.08g–i
	SG5	21.78ef	50.36h–j
	Salend	21.76ef	50.06i–k
July 6	SF1	20.01ij	52.34d–h
	SF2	20.71g–i	52.05e–i
	SF3	19.62jk	51.81f–i
	SK93	24.82a–c	54.98ab
	M13	24.24b–d	53.77a–f
	SG4	24.19b–d	52.79c–g
	SG5	23.99cd	53.51a–f
	Salend	22.32e	53.077b–g
July 21	SF1	21.28g	54.07a–d
	SF2	21.2g–i	53.77a–f
	SF3	20.28h–j	52.73c–g
	SK93	25.59a	54.38a–c
	M13	25.14ab	54.13a–d
	SG4	24.61bc	54.33a–d
	SG5	24.62a–c	55.22a
	Salend	24.44bc	54.86ab
LSD		1.31	2.37

*Note:* Means in each column, followed by similar letter(s) are not significantly different at the 5% probability level using the LSD test.

### 3.3 Protein Content

The results showed that the protein content was higher on 22 June (35.01%) compared to 6 July (33.35%) and 21 July (30.08%) (Table 8). The simple effect of genotype on protein content revealed that the highest (34.36%) and the lowest (30.47%) protein content appointed to Salend and SF1 genotypes, respectively (Table 8). Positive regression relationships were observed between protein content and both maximum temperature during seed‐filling (Figure 2a) and the length of the seed‐filling period (Figure 2b).

**TABLE 8:** Mean comparison of the simple effect of year, sowing date, and genotype on protein content and linolenic acid in 2017 and 2018.

	Protein content (%)	Linolenic acid (%)
Year		
2017	—	7.52a
2018	—	6.17b
lsd	—	0.38
Sowing date		
June 22	35.01a	5.99c
July 6	33.35b	6.88b
July 21	30.08c	7.66a
Genotype	1.08	0.47
SF1	31.79cd	7.33a
SF2	30.47d	7.44a
SF3	32.04b–d	7.1a
SK93	33.79ab	6.01c
M13	33.84a	6.79ab
SG4	33.14a–c	6.72a–c
SG5	33.08a–c	7.32a
Salend	34.36a	6.03bc
LSD	1.77	0.77

*Note:* Means in each column, followed by similar letter(s) are not significantly different at the 5% probability level using the LSD test.

### 3.4 Oleic Acid

**FIGURE 3:**
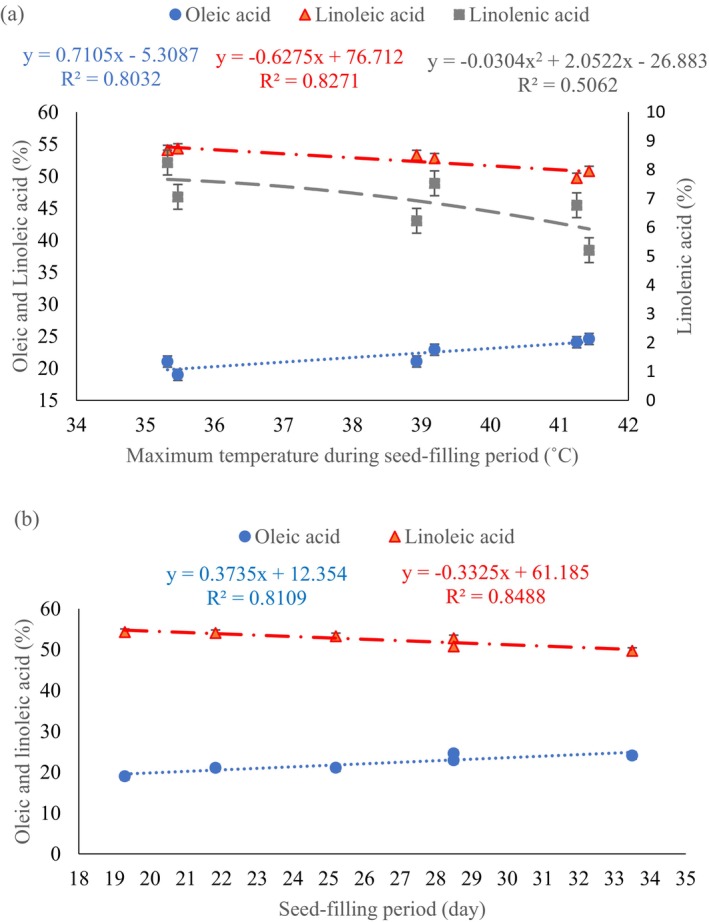
Regression relationship of oleic acid, linoleic acid, and linolenic acid with maximum temperature during the seed‐filling period (a) and oleic acid and linoleic acid with the length of the seed‐filling period (b). Means of interaction year × sowing date were used (each point shows mean three replications).

As can be seen in Table 6, the maximum oleic acid at the first sowing date (June 22) appointed to SK93 genotype in the first (29.06%) and second (31.31%) year. SK93 genotype had more oleic acid at all sowing dates compared to other genotypes (Table 6). The lowest level of oleic acid (18.53%) belonged to the SF1 genotype at the third sowing date (July 21) in the first year, while the lowest level (17.72%) was found in the SF2 genotype at the second sowing date (July 6) in the second year (Table 6). A positive relationship was observed between oleic acid content and both maximum temperature during seed‐filling (Figure 3a) and the length of the seed‐filling period (Figure 3b). The response of soybean genotypes was different in terms of oleic acid, so that SK93 produced the maximum (26.23%) and SF1 and SF2 genotypes had the minimum values of oleic acid during 2 years of the experiments (Figure 5b).

### 3.5 Linoleic Acid

Analysis of the interaction between sowing date and genotype on linoleic acid content across 2 years showed that all genotypes grown on July 21st exhibited higher linoleic acid levels compared to other sowing dates. The SG5 genotype achieved the highest linoleic acid content (55.22%) on July 21, while the lowest content was observed in SF3 genotype (June 22) (Table 7). In grain‐filling, there was a negative correlation between linoleic acid and maximum temperature, with higher temperatures enhancing linoleic acid and lowering it by 0.63% per degree Celsius (Figure 3a). Increasing the length of seed‐filling period led to decrease linoleic acid (Figure 3b). The highest percentage of linoleic acid observed in M13 (53.96%) and SF3 (50.93%) genotypes, respectively (Figure 5c).

### 3.6 Linolenic Acid

The average linolenic acid content was 7.52% in the first year and 6.17% in the second year (Table 8). Delaying the sowing date led to increase in linolenic acid. The highest (7.66%) and the lowest (5.99%) linolenic acid belonged to the third (July 21) and first (June 22) sowing dates (Table 8). Among the genotypes, the maximum value of linolenic acid was dedicated to SF2, SF1, SG5, and SF3, averaging 7.44%, 7.33%, 7.32%, and 7.1%, respectively, and the lowest one (6.01%) was observed in SK93 genotype (Table 8). As temperature increased during grain‐filling, linolenic acid content declined with a negative quadratic relationship (Figure 3a), indicating that linolenic acid content peaked at 35°C and then declined. The mean comparison of simple effect of genotype on linolenic acid showed that SF1, SF2, SF3, and SG5 genotypes produced more linolenic acid compared to other genotypes and SK93 had the lowest linolenic acid during 2 years of the experiment (Figure 5d).

### 3.7 Palmitic Acid

**FIGURE 4:**
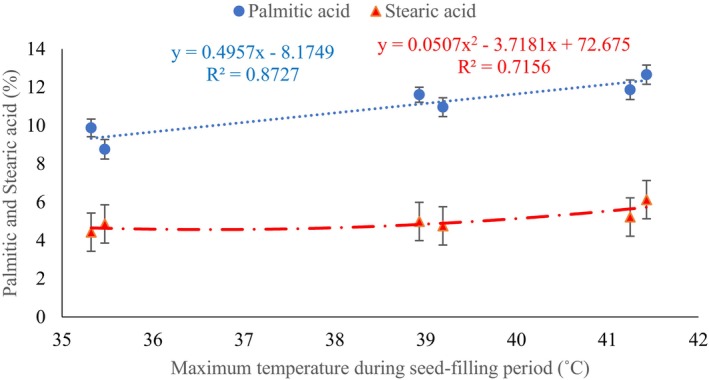
Regression relationship of stearic and palmitic acids with maximum temperature during the seed‐filling period. Means of interaction year × sowing date were used (each point shows mean three replications).

**FIGURE 5:**
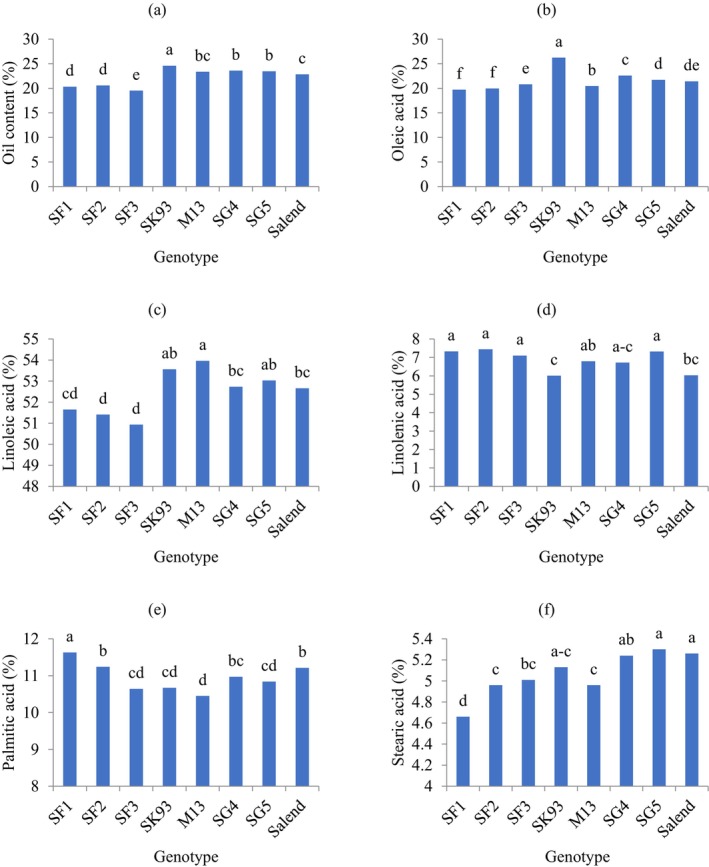
Mean comparison of oil content (a), oleic acid (b), linoleic acid (c), linolenic acid (d), palmitic acid (e), and stearic acid (f) of soybean genotypes during 2 years.

The palmitic acid content of genotypes responded differently to sowing dates in the 2 study years. In the first year, Salend genotype exhibited the highest palmitic acid content (13%) when sown on 22 June. As indicated in Table 6, however, in the second year, the SF1, SG5, and SF2 genotypes reached the highest values, reaching 13.34%, 13.22%, and 13.17%, respectively. Delaying sowing date caused a decrease of palmitic acid, and the lowest (8.88%) one was observed in Salend genotype on 21 July in the first year. The minimum palmitic acid (7.57%) was obtained in M13 genotype on 21 July in the second year (Table 6). The palmitic acid concentration enhanced linearly with increasing temperature during the grain‐filling period. A positive correlation (R2 = 0.87) was observed between palmitic acid and temperature (Figure 4). Mean comparison of palmitic acid in studied genotypes revealed that the maximum (11.63%) and the minimum (10.45%) corresponded to the SF1 and M13 genotypes, respectively (Figure 5e).

### 3.8 Stearic Acid

Soybean genotypes differed in their response to years in terms of stearic acid content. Therefore, the SF3, SK93, and SG5 genotypes had the highest values of stearic acid (5.49%, 5.43%, and 5.43%, respectively) in the second year and stearic acid content of the mentioned genotypes was more in the first year (Table 9). The lowest (4.2%) amount of stearic acid appointed to SF1 genotype in the first year (Table 9). Stearic acid enhanced quadratically by increasing temperature and reached the maximum amount at nearly temperature 41°C (Figure 4). Among genotypes, the highest (5.3%) and the lowest (4.66%) amount of stearic acid were related to the SG5 and SF1 genotypes, respectively (Figure 5f).

**TABLE 9:** Mean comparisons of the two‐way interaction of year × genotype on stearic acid during in each year of experiment (2017 and 2018).

Year	Genotype	Stearic acid (%)
First year (2017)	SF1	4.2e
	SF2	4.61c–e
	SF3	4.54de
	SK93	4.84b–d
	M13	4.84b–d
	SG4	5.13a–c
	SG5	5.15a–c
	Salend	5.11a–d
Second year (2018)	SF1	5.12a–c
	SF2	5.31ab
	SF3	5.49a
	SK93	5.43a
	M13	5.07a–d
	SG4	5.35ab
	SG5	5.43a
	Salend	5.41ab
LSD		0.57

*Note:* Means in each column, followed by similar letter(s) are not significantly different at the 5% probability level using the LSD test.

## 4 Discussion

Modeling experiments showed that temperature has an important effect on the reproductive stage of soybeans (Boote et al. 2018; Nguy‐Robertson, Suyker, and Xiao 2015), and a temperature higher than 30°C degrees will lead to a decrease in soybean yield (Boote et al. 2018). The study's findings highlight the significant effect of high temperatures during the soybean reproductive stage on quantitative and qualitative traits (Cohen et al. 2021; Iovane and Aronne 2022). Variations in temperature during grain‐filling directly affect oil composition (Carrera et al. 2017; Jianing et al. 2022). SYs varied greatly depending on different temperature conditions experienced over the 2‐year experiment. Elevated temperatures were directly linked to decreased SY, while increased seed‐filling periods did not lead to improved yields. This can be attributed to the higher temperatures experienced during the earlier sowing date (June 22) compared to the later sowing date (July 21). For every unit increase in seed‐filling duration, SY decreased by 57.27 kg.ha^−1^ (Figure 2b), emphasizing the stronger impact of temperature on yield compared to the seed‐filling period. Therefore, the extent of SY reduction was greater when the soybean plants were grown at a higher temperature.

The variability of soybean genotype responses to sowing dates demonstrates the importance of phenological adjustments in temperature adaptation. High temperatures during flowering can lead to pollen sterility and reduced SY (Bihter et al. 2017). As it can be seen in Tables 2 and 3, the duration of the reproductive stage and seed‐filling period for the genotypes under study in the first sowing date is longer than the second and third sowing dates. The number of pods per plant was 40, 55, and 60, respectively, on the dates of the first, second, and third sowing date (data not shown). First, sowing caused longer reproductive and seed‐filling periods, but these longer periods were not correlated with higher SYs. This suggests that temperature exerts a greater effect on SY than the duration of the seed‐filling period. Previous research showed that high temperatures can negatively affect pollen germination, pod number, seed number, and overall SY (Bihter et al. 2017; Alsajri et al. 2020; Nakagawa et al. 2020). Tolerant genotypes have good productivity and more SY under high temperatures (Staniak, Szpunar‐Krok, and Kocira 2023), and due to the fact that SK93 genotype had more SY at all sowing dates, it could be considered heat‐tolerant genotype.

The interaction between genotype and temperature during the seed‐filling period significantly affects protein content (Bellaloui et al. 2009; Bellaloui, Reddy, and Mengistu 2015). Earlier sowing date caused to coincide the seed‐filling period of genotypes to high temperature in the first sowing date during 2 years (Table 3), and all genotypes produced more oil in the third sowing date (Table 7). High temperatures limit the full realization of a genotype's genetic potential (Ahmad et al. 2021). This is attributed to factors, such as sugar restriction (Farooq et al. 2017), reduced photoassimilate availability for triacylglycerol biosynthesis (Pokharel et al. 2020), and decreased activity in the oil biosynthesis pathway (Iyer et al. 2008; Baud and Lepiniec 2010), ultimately leading to lower seed oil content. Regression analysis showed a negative relationship between oil content and maximum temperature during seed‐filling (Figure 2a) and the duration of seed‐filling (Figure 2b). However, protein content exhibited a positive relationship with these factors. For every 1°C increase in temperature during seed‐filling, oil content decreased by 0.34%, while protein content increased by 0.7% (Figure 2a). Similarly, for each unit increase in seed‐filling duration, oil content decreased by 0.18% and protein content increased by 0.41% (Figure 2b). The results indicate that oil accumulation at high temperatures is more sensitive than protein accumulation. Our results align with previous studies by Alsajri et al. (2020) and Song et al. (2016), demonstrating the differential effects of high temperature on oil and protein content. Temperature during the seed‐filling period profoundly influences the fatty acid profile of soybean (Hou et al. 2006; Alsajri et al. 2020). High temperatures affect the activity of enzymes involved in lipid biosynthesis, ultimately reducing oil synthesis (Elferjani and Soolanayakanahally 2018). The severity and duration of elevated temperatures play a crucial role in shaping fatty acid profiles (Kalantarahmadi and Daneshian 2023). Unsaturated fatty acids, such as oleic, linolenic, and linoleic acid, are particularly susceptible to temperature fluctuations, with notable variations observed across soybean cultivars. Delaying the sowing date and reducing seed‐filling temperatures (Table 3) led to lower oleic acid levels across all genotypes over the 2 years of the study (Table 6). For each 1°C increase in temperature during seed‐filling, oleic acid content rose by 0.71%, while linoleic acid content decreased by 0.62% (Figure 3a). According to Table 6, SK93 genotypes showed the highest levels of oleic acid in both years' first sowings. This was associated with higher mean and maximum temperatures compared to other sowing dates (Table 3). Similar trends were observed in other genotypes (Table 6). Soybean genotypes showing stable oleic acid levels across various sowing dates and temperatures offer a promising source of favorable fatty acid profiles. Elevated temperatures during seed‐filling consistently led to higher oleic acid and lower linoleic acid, aligning with previous research (Alsajri et al. 2020; Carrera and Dardanelli 2017). While the length of the seed‐filling period did not significantly impact linolenic acid, oleic acid increased linearly with increasing duration (Figure 3b). Conversely, a negative relationship was observed between linoleic acid and seed‐filling duration (Figure 3b). For each unit increase in seed‐filling duration, oleic acid increased by 0.37%, and linoleic acid decreased by 0.33% (Figure 3b). Genetic characteristics and temperature during seed‐filling play a pivotal role in shaping oil composition (Bellaloui, Reddy, and Mengistu 2015). Previous studies confirmed that high temperatures lead to reduced linoleic and linolenic acid levels, while increasing oleic acid (Dornbos and Mullen 1992; Bellaloui, Mengistu, and Kassem 2013; Alsajri et al. 2020). Fatty acid profiles may be influenced by temperature by enzymes that control fatty acid conversion and accumulation (Bachlava and Cardinal 2009). Grace et al. (2007) reported that the expression level of genes (SAD‐B, SAD‐A, and FAD2‐1) effective in the synthesis of oil and fatty acids decreases at high temperature (26/30°C) compared to the optimal temperature (22/26°C) and the decrease in expression FAD2‐1 gene increased oleic acid and decreased linoleic acid in soybean genotypes at high temperature. Furthermore, reducing the expression of SAD genes at high temperature increased the amount of stearic acid.

Soybean oil in an edible oil and different genotypes have various fatty acid profiles. Therefore, identifying the genotypes that have more unsaturated fatty acids can be a step toward improving the quality of the oil to improve the health of the society. The concentration of unsaturated fatty acids ranges nearly 24% for oleic, 54% for linoleic, and 8% for linolenic acids. The amount of saturated acids is about 10%–12% and 3%–5% for palmitic and stearic acids, respectively (Bellaloui, Reddy, and Mengistu 2015). The increase in oleic acid levels can be attributed to a reduced conversion of oleic acid into linoleic acid under elevated temperatures (Dornbos and Mullen 1992). This conversion process is regulated by the enzyme oleate desaturase, and fluctuations in the oleic‐to‐linoleic acid ratio during drought and heat stress are likely influenced by its activity (Flagella et al. 2002).

Palmitic and stearic acids are major saturated fatty acids (Badawy et al. 2016), and their levels in soybean are influenced by temperatures during seed‐filling (Alsajri et al. 2020). While palmitic acid exhibited a linear relationship with maximum temperature during seed‐filling (Figure 4), stearic acid displayed a polynomial trend. The study found that delaying the sowing date resulted in lower palmitic acid content (Table 6). As observed in Table 3, all genotypes' seed‐filling periods experienced higher temperatures during the 22 June sowing, as compared to the 21 July sowing, and palmitic acid levels decreased as sowing dates advanced.

**FIGURE 6:**
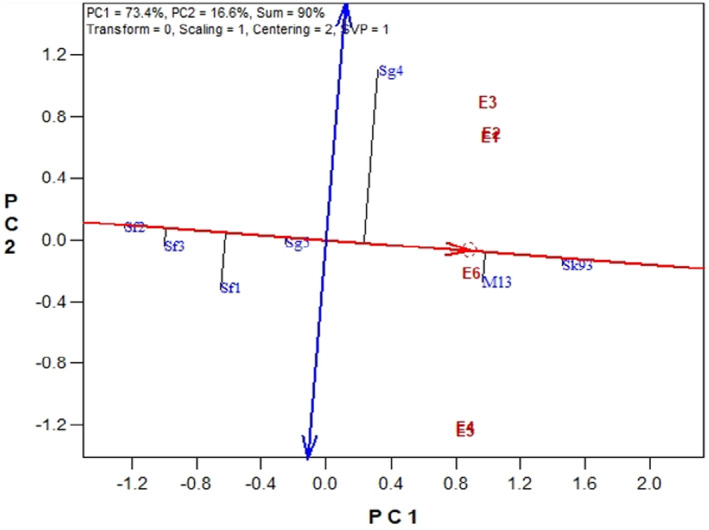
The average GGE tester biplot for seed yield stability of soybean genotypes. E1, First sowing date in the first year; E2, Second sowing date in the first year; E3, Third sowing date in the first year; E4, First sowing date in the second year; E5, Second sowing date in the second year; E6, Third sowing date in the second year.

The results of the stability analysis using the GGE biplot method revealed that the SK93 and M13 genotypes exhibited high SY (Figure 6). In this method, a genotype located toward the right end of the horizontal axis of the graph indicates higher yield, while a genotype positioned toward the left end indicates lower yield (Yan et al. 2007). Consequently, the SF2 and SF3 genotypes showed the lowest SY. Moreover, each genotype is marked with a dashed line extending toward the vertical axis, indicating the stability of the genotypes across three different sowing dates over 2 years of experimentation. Here, a shorter height of the dashed line implies higher stability, while a greater height indicates lower stability (Okello‐Anyanga et al. 2016). There is a vertical axis that passes through the coordinate axis perpendicular to the mean environment axis, indicated by two arrows. This axis represents the interaction effect of genotype in the environment and determines genotype stability. The higher the genotype cluster on this axis (regardless of direction), the more unstable they are. Using this biplot, it was evident that genotypes SK93, SF2, and SG5 exhibit greater stability compared to other genotypes. Genotypes SG4 and SF1 were identified as the least stable genotypes. In general, considering yield and stability is essential for different environments; therefore, genotype SK93 can be selected for simultaneous high yield and stability.

## 5 Conclusion

This study assessed the effect of temperature on SY, oil content, protein content, and fatty acid composition in soybean genotypes. The results highlight the significant influence of temperature during the reproductive stage and seed‐filling period on these critical traits. The study found that SY, protein content, oil content, and fatty acid composition varied across soybean genotypes in response to different sowing dates. Increased temperatures during seed‐filling led to a linear increase in protein and oleic acid content, while oil and linoleic acid content decreased linearly. Temperatures exceeding 36°C during seed‐filling significantly reduced SY. SK93 genotype consistently demonstrated superior seed production, oil content, and oleic and linoleic acid levels across all sowing dates, establishing it as the most stable and adaptable genotype. Considering soybean traits' sensitivity to temperature, it is vital to select genotypes that are tolerant to elevated temperatures. Based on obtained results and the superiority of SK93 genotype in different temperature conditions, this genotype can be used in the advancement of breeding programs to improve the adaptability and performance stability of other genotypes.

## Conflicts of Interest

The authors declare no conflicts of interest.

## Data Availability

The authors have nothing to report.
